# Alpha‐2a adrenergic receptor activation in genetic absence epilepsy: An absence status model?

**DOI:** 10.1002/epi4.12879

**Published:** 2024-01-18

**Authors:** Melis Yavuz, Serdar Akkol, Filiz Onat

**Affiliations:** ^1^ Department of Pharmacology, Faculty of Pharmacy Acibadem Mehmet Ali Aydinlar University University Istanbul Turkey; ^2^ Department of Neurology University of Alabama at Birmingham Medical Center Birmingham Alabama USA; ^3^ Department of Medical Pharmacology, School of Medicine Marmara University Istanbul Turkey; ^4^ Department of Medical Pharmacology, School of Medicine Acibadem Mehmet Ali Aydinlar University Istanbul Turkey

**Keywords:** absence status epilepticus, alpha‐adrenergic receptors, dexmedetomidine, GAERS, genetic absence epilepsy

## Abstract

**Objective:**

The objective of the study was to propose a candidate animal model of absence status epilepticus induced by specific alpha‐2a adrenergic receptor (α2AR) activation. We also aim to investigate the responsiveness of this model to classical anti‐status or anti‐absence medications.

**Methods:**

An α2AR agonist, dexmedetomidine (DEX), was injected intracerebroventricularly into adult rats with genetic absence epilepsy, and their electroencephalography (EEG) was recorded. The total duration, number, and mean duration of each spike‐and‐wave discharges (SWDs) were evaluated. The blocks of absence status events were classified as the initial and second sets of absence statuses. Ethosuximide (ETX) was administered as a pretreatment to another group of rats and later injected with 2.5 μg DEX. In addition, ETX, valproic acid (VPA), diazepam (DIAZ), and atipamezole (ATI) were administered after induced status‐like events following DEX administration. Power spectral characteristics and coherence analysis were performed on the EEG to assess the absence status events and sleep.

**Results:**

The 2.5 μg dose of DEX increased the total SWD duration and induced continuous SWDs up to 26 min. Following the initial absence status event, sleep was induced; then, the second period of absence status‐like activities were initiated. ETX pretreatment blocked the occurrence of absence status‐like activities. Power spectral density analyses revealed that DEX‐induced post‐sleep activities had higher power in delta frequency band (1–4 Hz) and attenuated power of 7 Hz harmonics (14 and 21 Hz) than the pre‐injection seizure. The mean duration of SWDs were decreased in all the groups, but occasional prolonged activities were seen in ETX or VPA‐injected rats but not with DIAZ or ATI.

**Significance:**

This study presents an absence status epilepticus animal model that is activated by α2AR activation to investigate the pathophysiological role of absence status. Unlike other agents ATI switched off the second set of absence statuses to normal SWDs, without sedation or lethargy, can show it may preferentially block absence status‐like activity.

**The Plain Language Summary:**

This study proposes a rat model for prolonged seizures, resembling absence status epilepticus. Activating the brain’s alpha‐2a adrenergic receptor with dexmedetomidine induced seizures lasting up to 26 minutes. Ethosuximide pretreatment and post‐treatment with valproic acid, diazepam, and atipamezole decreased induced seizures. The findings suggest this model is valuable for studying absence status epilepticus. In addition, atipamezole normalized abnormal seizures without sedation, hinting at its potential for targeted treatment and further research.

**Key Points**

- This study presents an acute absence status epilepticus model with specific α2AR activation.
- α2AR activation induces prolonged spike‐and‐wave discharges and also proposes a model to study modification of SWDs.
- Caution is necessary for the clinical usage of anesthetics such as DEX in patients with epilepsy.
- Atipamezole (ATI), an α_2AR_ blocker, suppressed the two phase of events induced by DEX, can be a candidate for absence status epilepticus.

## 1 INTRODUCTION

Status epilepticus is a highly fatal condition with an incidence of 0.02% and is associated with neuropathological changes.^1^, ^2^, ^3^ Although, convulsive status epilepticus has been classified, the treatment regimens follow specific guidelines, and the non‐convulsive status epilepticus (NCSE) has only been recently characterized with a revised definition.^4^ NCSE consists of up to 63% of patients with status epilepticus^1^, ^5^ and 25% of those with focal status epilepticus.^6^ NCSE can even be observed in 8% of comatose patients without clinical seizure activity.^7^ Recently, the induction of a single non‐convulsive seizure has been shown to cause pathophysiological changes.^8^ Absence status epilepticus^9^ is defined as the duration of >10 min and is continuous without any prominent motor phenomena^4^ that differentiate absence status from other NCSEs. Although not clearly defined, absence status can be expected to represent certain characteristics of the NCSE. NCSEs are defined as “focal or generalized spike‐and‐discharges (SWDs) below 2.5 Hz, which is slightly less than an average SWD frequency or rhythmic waves at >0.5 Hz (theta‐delta) with (a) incrementing onset (increase in voltage with increase or decrease in frequency), (b) evolution in pattern (increase or decrease in frequency) (>1 Hz) or location, (c) decrementing termination (voltage or frequency), or (d) post‐periodic epileptiform discharges background with slowing or attenuation 9.” As a result absence status is still to be understand, but can be expected to share similar electrophysiological characteristics as other spike‐and‐wave discharges and slower in frequency. To better understand and characterize absence status epilepticus, animal models are needed.

Few studies proposed animal models of NCSE^8^, ^10^, ^11^; although many studies report motor seizure models.^12^, ^13^, ^14^, ^15^, ^16^ Other studies report continuous seizures in rats with petit‐mal‐like seizures, the ancestors of Genetic Absence Epilepsy Rats from Strasbourg (GAERS), induced by administering mixed antagonists of dopamine D1/D2 receptors.^17^ Other specific uses of agonists or antagonists on dopamine (D1) or D2 receptors did not have such effects as in Warter et al.'s study.^17^ Buzsaki et al.^18^ reported a significant increase in high‐voltage spindles with clonidine, another alpha‐2a receptor agonist (α_2AR_), in 344 Fischer rats. In 1994, Inoue et al.^19^ reported nearly continuous SWDs by administering a combination of a dopamine antagonist and fentanyl. Duysens et al.^20^ also reported nearly continuous SWD following a non‐anesthetic dose of etomidate, with morphological changes in SWDs by slowing down the intra‐spike frequency. This finding may be comparable to that of the current study. However, none of these studies have induced an actual continuous SWD pattern that can be classified as an absence status epilepticus without any motor component and long continuous seizures with the SWD morphology.^2^

Based on our previous findings suppressing the SWD activity of atipamezole (ATI), an α_2AR_ blocker,^21^ we expected a specific agonist of α_2AR_ dexmedetomidine (DEX) might increase the SWD activity. Previously we have shown that ATI suppresses the SWDs at the dose of 12 μg.^21^ In the literature, 1 to 5 times higher doses of ATI were used to recover DEX‐induced anesthesia or sedation^22^, ^23^ and DEX induces sedation above the doses of 3 μg and also induces high‐voltage spindles.^22^ Although a study addressed that DEX can prolong SWDs via intraperitoneal route as well,^24^, ^25^, ^26^ we picked the dose of 2.5 μg 1/5 of ATI dose as well as the doses slightly below the sedative doses in order to understand if DEX has an effect on the SWDs expressed in GAERS, as well as to antagonize ATI. In our previous study, as we established the SWD‐suppressing dose of ATI through intracerebroventricular (i.c.v.) route, we administered 2.5‐μg DEX in the GAERS model, to reverse that effect. We used the same method of drug administration in this study as well to be able to follow through experiments since we optimized this method previously. As a result, a continuous SWD activity that was established over 1 min was considered as an acute absence status. DEX‐induced three phases of events. Immediately after injecting a single 1–2‐min‐long continuous SWD was triggered, followed by anesthesia‐induced sleep for approximately 40 min. When the rats were awake from anesthesia, the second period of absence status(es) started. In summary, this study, the block of SWD events that may represent an acute absence status epilepticus in GAERS was characterized and evaluated. We also investigated the effects of a classical anti‐absence drug ethosuximide (ETX), and anti‐status drugs; valproic acid (VPA), and diazepam (DIAZ), as well as the specific α_2AR_ antagonist ATI on these induced‐absence status‐like activities in order to assess whether this model will respond to classical known drugs used against absence seizures or status epilepticus. We propose the specific activation of α_2AR_ may trigger absence status in genetic absence epilepsy and the α_2AR_ antagonists are potential candidates for absence status therapy.

## 2 METHODS

### 2.1 Animals

In the experiments, male GAERS rats (age, 3–4 months; weight, 250–350 g) known to have spontaneous SWD activities were used. They were obtained from the breeding colony of the Acibadem Mehmet Ali Aydinlar University Experimental Animals Unit. The rats were housed in a temperature‐controlled room (21 ± 3°C) and a 12‐h light/dark cycle (lights on at 8 a.m.). Rats were individually placed in a cage and provided ad libitum food and water. All procedures performed on rats were approved by the Ethical Committee for Experimental Animals of Acibadem Mehmet Ali Aydinlar University (Protokol no: 071.2018.mar and 2023/48) and conform with the EU Directive 2010/63/EU for animal experiments and ARRIVE guidelines.

**FIGURE 1:**
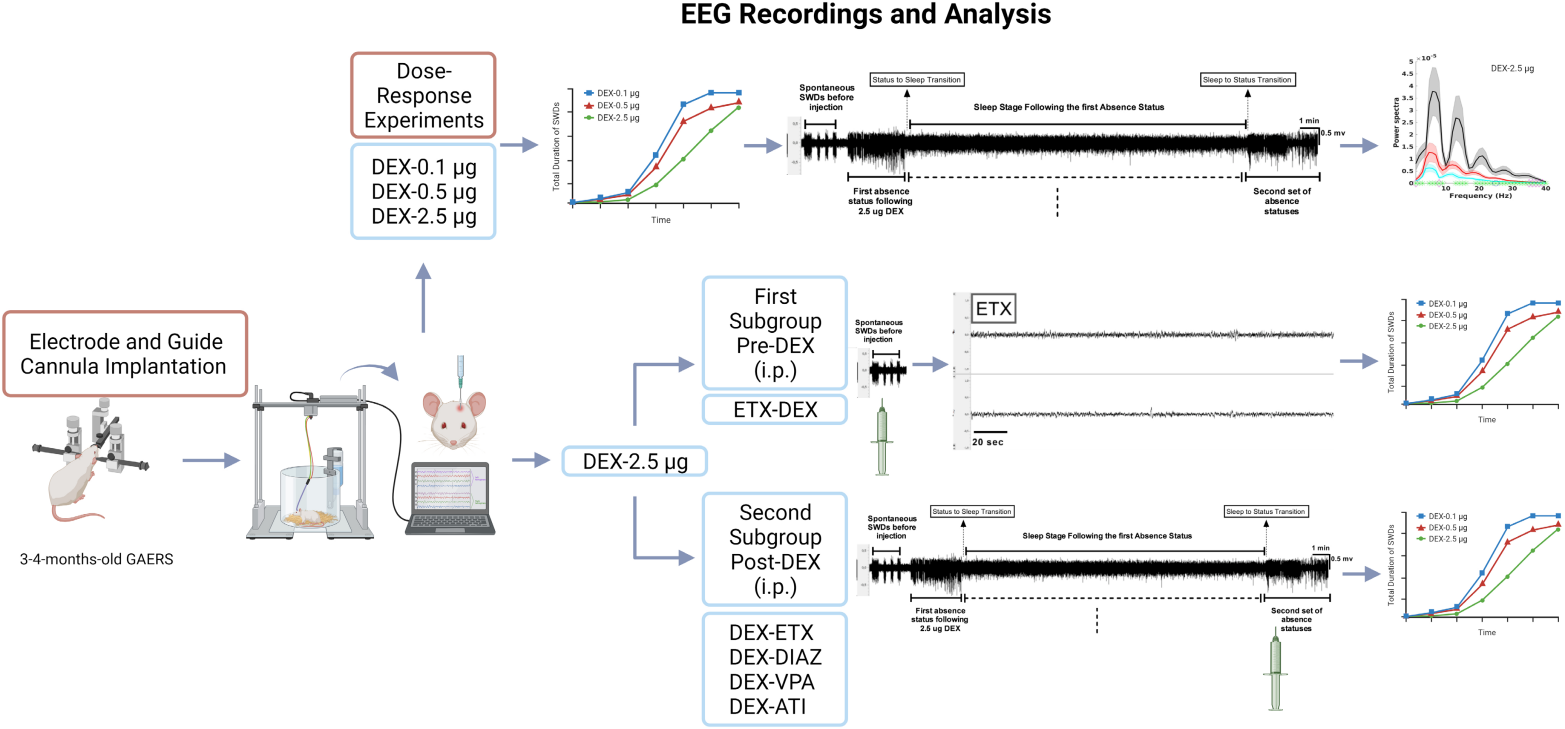
Scheme of experiments: DEX: Dexmedetomidine, ETX: Ethosuximide, DIAZ: Diazepam, VPA: Valproic acid, ATI: Atipamezole, EEG: Electroencephalography, GAERS: Genetic Absence Epilepsy Rats from Strassbroug. This methodology schema outlines the key steps and procedures followed in the study to assess the effects of DEX and other drugs on the SWD activities generated by DEX in 3–4‐months‐old‐GAERS rats. GAERS were implanted with the cortical electrodes and guide cannula (i.c.v.). In the dose–response experiments DEX was administered i.c.v. at different doses (0.1 μg, 0.5 μg, and 2.5 μg), and control groups received aCSF and SWDs were analyzed. The spectral characteristics and coherence analysis were performed on the EEG of GAERS in which the absence status like activities were induced by 2.5 μg DEX. Sleeping time and absence status classification were also performed on this group. In another set of experiments, after inducing absence status‐like activities with 2.5 μg i.c.v. DEX, we tested whether this induced status would respond to classical drugs known for their efficacy against absence seizures or anti‐status drugs: ETX, and anti‐status drugs; VPA, and DIAZ, as well as the specific α2AR antagonist ATI, which were administered via intraperitoneal injections post‐DEX at the beginning of the second period of absence statuses (Second Subgroup Post‐DEX). ETX was also administered pre‐DEX to determine if the activity would be halted (First Subgroup Pre‐DEX).

Rats were randomly assigned to evaluate the dose‐dependent effects of DEX, and the number of rats was determined based on our previous studies.^21^ Groups receiving i.c.v. DEX with doses of 0.1, 0.5, and 2.5 μg were designated as DEX‐0.1 μg, DEX‐0.5 μg, and DEX‐2.5 μg, respectively. The baseline recording data of rats and those that received artificial cerebrospinal fluid (aCSF) were labeled as CONT‐Basal and CONT‐aCSF, respectively. The group of rats administered 200 mg/kg of ETX intraperitoneally at 20 min before the i.c.v. injection of DEX was labeled as ETX‐DEX‐2.5 μg, whereas those administered with 200 mg/kg of ETX, 5 mg/kg of DIAZ, 200 mg/kg VPA and 1 mg/kg ATI that have been administered intraperitoneally immediately after initiating DEX (after sleep) were labeled as DEX‐ETX, DEX‐DIAZ, DEX‐VPA, and DEX‐ATI, respectively. The EEG was recorded, and the total duration, the mean duration of each seizure episode, and the frequency of seizures were evaluated in all these groups. In addition, the power spectral and coherence analyses, and the characterization of the block of SWD events were performed to evaluate the sleeping time and absence status activities on the EEG data in the DEX‐2.5 μg group (Figure 1).

### 2.2 Drugs

The specific α_2A_AR agonist DEX and aCSF were purchased from (Tocris 2749; 3525). Three different doses of DEX (0.1, 0.5, and 2.5 μg) were dissolved in the aCSF. The control group received aCSF in the same volume as the drug (5 μL). The drugs and the vehicle were administered i.c.v., and ETX, DIAZ (DEVA Pharmaceuticals), VPA (Depakin Chrono BT 500 mg), and ATI^21^ were dissolved in saline and injected intraperitoneally (Figure 1).

### 2.3 Stereotaxic surgery

All experimental and control groups of rats were anesthetized with ketamine (100 mg/kg IP, Alfamine %10; Alfasan International B.V.) and xylazine (10 mg/kg, IP, Alfazyne %2; Alfasan International B.V.). Each animal was placed in a stereotaxic cage (Stoelting Model 51 600, Stoelting Co., Illinois, USA). A longitudinal incision was made over the skull, and four stainless steel screws with specifically made insulated wires were implanted bilaterally over the frontoparietal cortex for cortical EEG recordings. The electrodes were connected by insulated wires to a micro‐connector for EEG recordings. A guide cannula was implanted in the lateral ventricle at the coordinates (AP, −1.0 mm; ML, −1.4 mm; and V, −4.1 mm) according to Paxinos and Watson.^27^ The electrodes and wires were covered by dental acrylic and fixed to the skull.

### 2.4 EEG recordings and analysis

#### 2.4.1 Intracerebroventricular injection of DEX, EEG recordings, and SWD evaluation

After the electrodes and cannulas were implanted by stereotaxic surgery, the rats were allowed to rest for a week. After a 1‐week recovery, a 3‐h baseline activity of CONT‐Basal rats was recorded. On the next day, a 40‐min baseline EEG activity was recorded in the EEG groups. After the 40‐min recording, while the control GAERS (CONT‐aCSF) were injected with 5 μL of aCSF, the drug groups received DEX dissolved at different doses in the aCSF (DEX‐0.1 μg, DEX‐0.5 μg, and DEX‐2.5 μg). After the i.c.v. injections of DEX, 3‐h EEG recordings were evaluated. SWD complexes were analyzed, which are generally identified if their duration was >1 s, with a train of sharp wave followed by a slow wave (7–11 Hz) with amplitude of at least twice the background amplitude of the EEG. Criteria between two successive SWDs was minimum 1 s. EEG was amplified through a BioAmp ML 136 amplifier, with band‐pass filter settings of 1–40 Hz, recorded and analyzed using the Chart v7 program (PowerLab8S ADI Instruments, Oxfordshire, UK).

#### 2.4.2 ETX, DIAZ, VPA, and ATI injections and SWD evaluation

One week after the stereotaxic surgery, a baseline EEG activity of rats in the ETX‐DEX‐2.5 μg group was recorded for 20 min. Then, the rats were pretreated with 200 mg/kg of ETX intraperitoneally 20 min before intracerebroventricularly injecting 2.5 μg of DEX, the dose that has been previously established to induce the status events as in this study. The dose of ETX was higher than the previously established doses to suppress the SWD activity^28^, ^29^, ^30^ and prevent absence status. Likewise, following a baseline EEG activity of rats in the DEX‐ETX, DEX‐DIAZ, DEX‐VPA, and DEX‐ATI groups, After the i.c.v. injections, EEG recordings were performed for 3 h. SWD complexes were analyzed as explained above.

#### 2.4.3 Power spectral and coherence analyses of EEG recordings

The power spectral density and coherence differences were compared among long seizures (>20 s) before injecting 2.5 μg of DEX (DEX‐2.5 μg) (pre‐injection‐basal SWD activities), the seizure(s) after the injection (post‐injection—initial induction of absence status), and seizures occurring after the rats woke up from the DEX‐induced sleep (post‐sleep‐second period of absence statuses) recorded from the same recording day. Non‐artifactual periods recorded from six rats were excluded. Multitaper spectral decomposition was used at 1‐Hz frequency steps with discrete prolate spheroidal sequences and 2‐Hz multitapers between 1 Hz and 40 Hz. For coherence, the following formula was used:

$$\left|C_{\mathrm{ij}}\left(f\right)\right|∶=\left|\frac{\left\langle A_{i}\left(f\right)A_{j}\left(f\right)e^{t∆\theta_{\mathrm{ij}}\left(f\right)}\right\rangle }{\sqrt{\left\langle A_{i}^{2}\left(f\right)\right\rangle \left\langle A_{j}^{2}\left(f\right).\right\rangle }}\right|$$

where i and j are recordings from the right and left hemispheres, $A_{i}\left(f\right)$ and $\theta_{i}\left(f\right)$ represent the amplitude and phase values calculated from the complex output of the time–frequency decomposition at a frequency *f*.^31^ The power spectral density (PSD) was defined as the amplitude of the time–frequency decomposition calculated for each frequency. For each animal, a single status event was chosen for each type of activity while blinded to the type and selected the activity without electrical interference. Thus, we analyzed PSDs of 5‐s epochs of each animal and of each type of status. All analyses on PSD and coherence were conducted using Fieldtrip toolbox^32^ on MATLAB software (R2019a, MathWorks, Natick MA, USA).

### 2.5 Sleeping time and absence status classification

After injecting 2.5 μg of DEX (DEX‐2.5 μg), all rats had a single or two 1–2‐min‐long continuous SWD, which was classified as the initial absence status as discussed in this study. Following that event(s), sleep was induced for approximately 40 min, and the mean duration and onset of DEX‐induced sleep were calculated. The block of continuous SWD events after terminating sleep was classified as the second period of absence status(es). The status events were defined as continuous and over 1 min prolonged SWD activities. They were selected if their duration is >1 min and classified based on their durations: <1 min, 1–2 min, and >5 min.

### 2.6 Histological verification of i.c.v. injections

All groups that received i.c.v. injections were verified histologically. The rats were anesthetized with ketamine (100 mg/kg) and xylazine (10 mg/kg) intraperitoneally. The 5‐μl volume of 1% methylene blue with the same amount of drugs administered for the experiments, was injected into the right lateral ventricle. The internal was kept at the injection site for approximately 60 s before removal. The rats were decapitated, and the brains were isolated. Methylene blue traces were examined in the ventricles to confirm the i.c.v. injection locations.

### 2.7 Statistical analysis

All statistical analyses were performed with GraphPad Prism version 9.00 (GraphPad Software, San Diego, USA). To statistically compare the EEG data in DEX‐injected groups, a two‐way analysis of variance (ANOVA) followed by Dunnett's post hoc test was performed. To statistically compare the ETX effects in the injection groups, two‐way ANOVA followed by Tukey's post hoc test was performed. A difference of *p < 0.05 was considered statistically significant. To statistically compare PSD and coherence, Mann–Whitney *U* test was performed between each seizure at each frequency and then corrected for false discovery rate with a *q*‐value of 0.01.^33^

## 3 RESULTS

### 3.1 Average time for the initial induction of absence status and sleep

**FIGURE 2:**
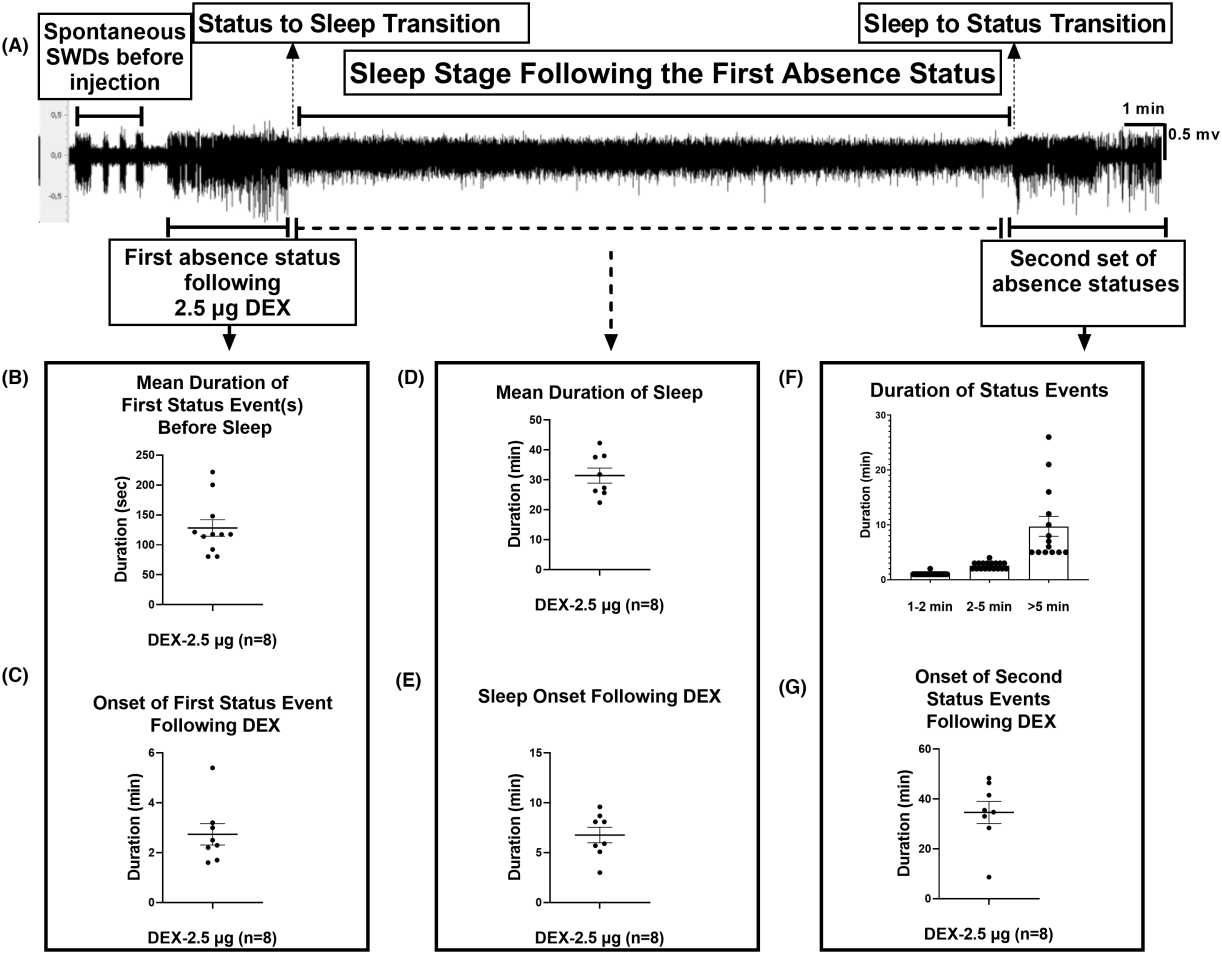
Events after the DEX injection: The first absence status(es)–sleep–second set of absence statuses. DEX: Dexmedetomidine and DEX‐2.5 μg: 2.5 μg i.c.v. DEX injection. (A) The EEG pattern before and after the 2.5 μg DEX injection. Before the injection, 5–20 s of the SWD activity is observed on EEG. Seconds after the injection, an absence status epilepticus event is observed, followed by approximately 40 min of sleep. After the sleep stage, multiple and continuous absence status epilepticus events, namely, the second set of absence statuses, occur. The second set of absence statuses varies between 1 and 26 min. (B) The mean duration of the first absence status after injecting 2.5 μg of DEX, varies between 1 and 4 min. (C) The onset of the first absence status event shows the first absence status that occurs 1–5 min post‐injection. (D) The mean duration of sleep induced by 2.5 μg DEX between 20 and 42 min. (E) The onset of sleep occurs immediately after the first absence status event. (F) The classification of the second set of absence status events. The events are classified as 1–2 min, 2–5 min, and >5 min. The mean duration can be observed in the chart. (G) The onset of the second set of absence status events after the DEX injection.

Following the i.c.v. injection of 2.5‐μg DEX, a specific EEG pattern was observed in all rats (DEX‐2.5 μg group; *n* = 8; Figure 2A). The first event was a continuous seizure with a duration of either 1–2 min or 2–5 min, which is much longer than a typical SWD. The pattern of this event was similar to that of SWDs. Therefore, this event and the following continuous SWD events over 1 min were classified as an absence status epilepticus. The average duration of the initial absence status was 128 ± 13.74 min (Figure 2B). The onset time of this event after the injection was 2.74 ± 0.42 min (Figure 2C) among all rats. Following that initial absence status event(s), all rats had an anesthesia‐induced sleep for approximately 31.43 ± 2.53 min (Figure 2D), with a latency of 6.78 ± 0.78 min after the DEX injection (Figure 2E).

### 3.2 Characterization of the second period of absence statuses

**FIGURE 3:**
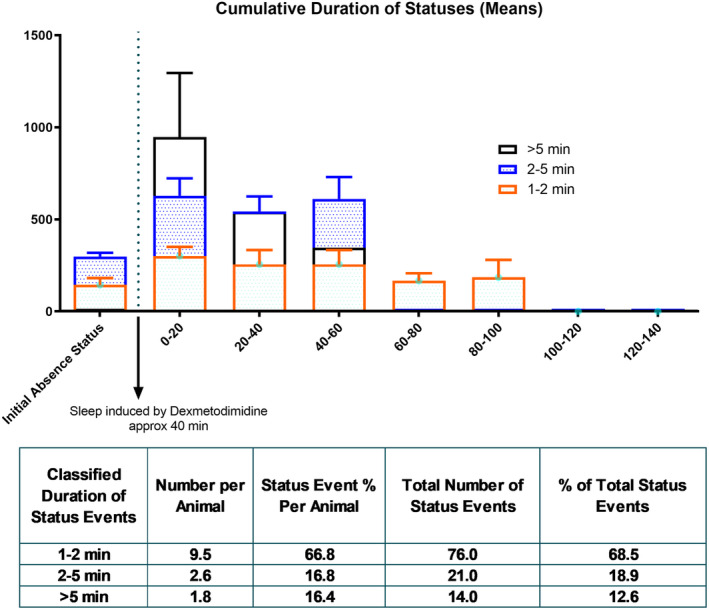
Characterization of the second set of absence status epilepticus. DEX: Dexmedetomidine. In the graph, the distribution of absence status events classified as 1–2 min, 2–5 min, and >5 min is observed on the time‐course after the DEX injection. Clustered events on the first 0–20 min before the break panel represents the in‐between sleep state, the first absence status epilepticus events. It is observed that the first set does not have status activity for >5 min. The table shows the means of the number of events per animal, status events as percentages, total number of status events, and % of total status events of each classified second set of absence status events.

Immediately after the cessation of sleep in the DEX‐2.5 μg group, the second period of absence status(es) was initiated and characterized as follows: 1–2 min, 2–5 min, and >5 min. The overall mean duration of all second period of absence status(es) was 170.1 ± 23.02 s. All rats (100%) had absence status events after the DEX injection and the sleep phase. During the second period of absence status(es) of all rats (*n* = 8), 70, 17, and 14 events occurred during the 1–2‐, 2–5‐, and >5‐min seizures were counted (Figure 3).

### 3.3 Effects of DEX on SWD parameters

**FIGURE 4:**
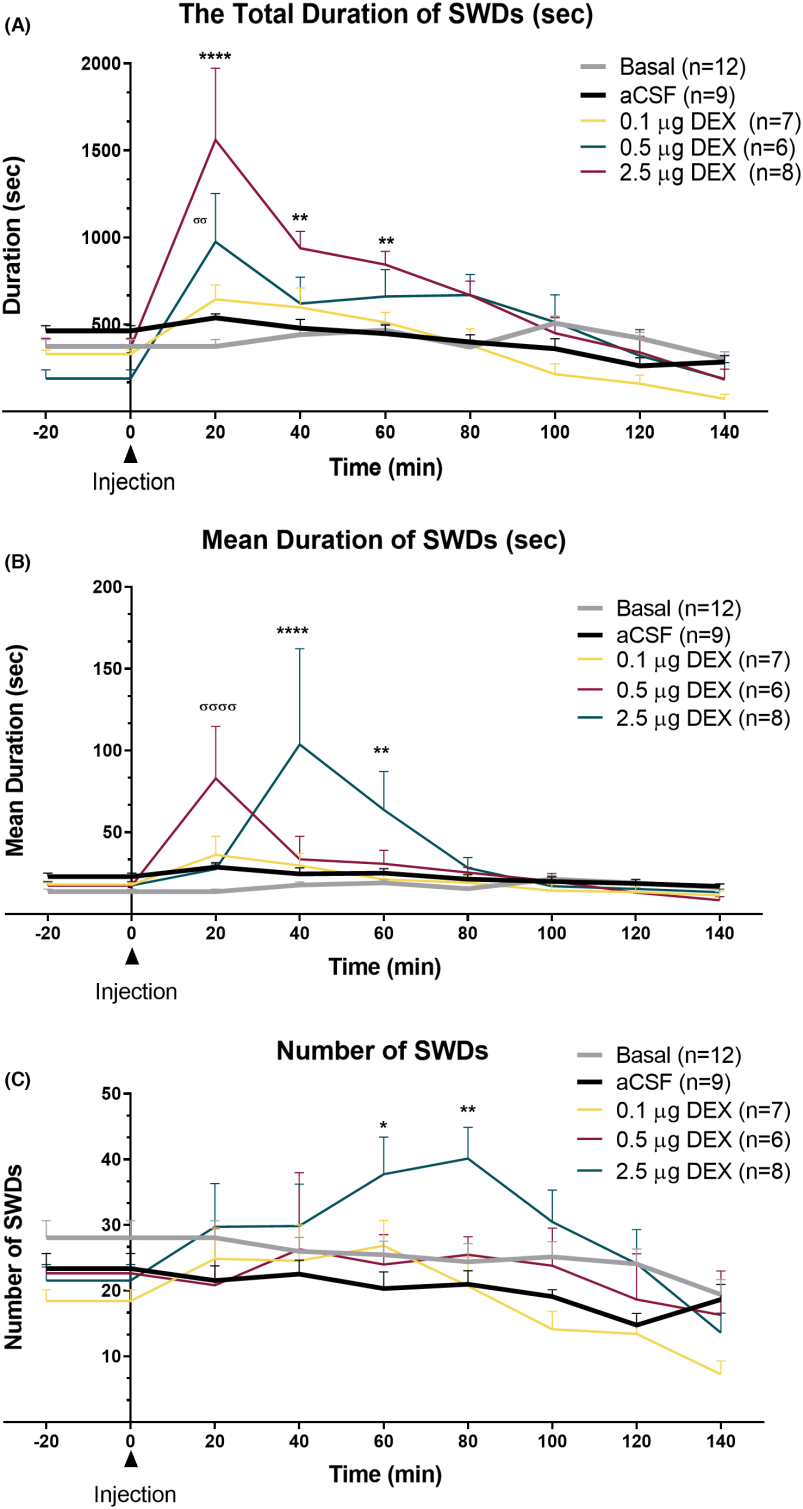
Effects of acute injection of DEX on SWD parameters in GAERS (aCSF control vs. DEX). SWDs: Spike‐and‐wave discharges, the dose of 0.1 μg: DEX‐0.1 μg, 0.5 μg: DEX‐0.5 μg, 2.5 μg: DEX‐2.5 μg. (A) The mean total duration of SWDs in different doses of i.c.v. administered DEX. The dose of 0.1 g Data are given as means ± SEM (*p < 0.05; two‐way ANOVA followed by Dunnett's post hoc test showed a significant increase with doses of 0.5 and 2.5 μg). Time 0 indicates the time of DEX injection. The number of rats is given in the legends. (B) The number of SWDs in different doses of i.c.v. administered DEX. (C) The mean duration of one SWD complex, the ratio of total SWD duration to the number of SWDs (*p < 0.05; the significant increase at doses of 0.5 and 2.5 μg of DEX).

The total duration of all SWDs and absence status activities in the DEX‐0.5 μg group at 20 and in the DEX‐2.5 μg group at 20‐, 40‐, and 60‐min time points were significantly greater than that in the CONT‐aCSF group (“Time” effect: *F* (8, 333) = 21.11, *p* < 0.001; “Treatment” effect: *F* (4, 333) = 12.47, *p* < 0.001; Interaction of “time” and “treatment”: *F* (32, 333) = 4.17, *p* < 0.001) (Figure 4A). The time points represent the point approximately 45 min (see average sleep duration) after DEX administration when the rats were awakened from the DEX‐induced anesthesia. The frequency of seizures was also significantly increased with 2.5 μg of DEX (DEX‐2.5 μg), (“Time” effect: *F* (8, 330) = 4.851, *p* < 0.001; “Treatment” effect: *F* (4, 33) = 10.50, *p* < 0.001) without any significant interaction between time and treatment (Figure 4B).

Moreover, the mean duration of each SWD was significantly increased in both DEX‐0.5 μg and DEX‐2.5 μg groups. In the DEX‐0.5 μg group at 20 min and in DEX‐2.5 μg at 40 and 60 min, the duration of seizures was increased (“Time” effect: *F* (8, 331) = 2.198, *p* < 0.001; “Treatment” effect: *F* (4, 331) = 4.081, *p* < 0.001; Interaction of “time” and “treatment”: *F* (32, 331) = 2.19, *p* < 0.001) (Figure 4C). The SWDs showed a continuous pattern; therefore, these episodes were classified as absence status epilepticus(es), if the interval between them did not reach to 1 s.

### 3.4 ETX pretreatment to determine the effects of DEX on the DEX‐induced events

**FIGURE 5:**
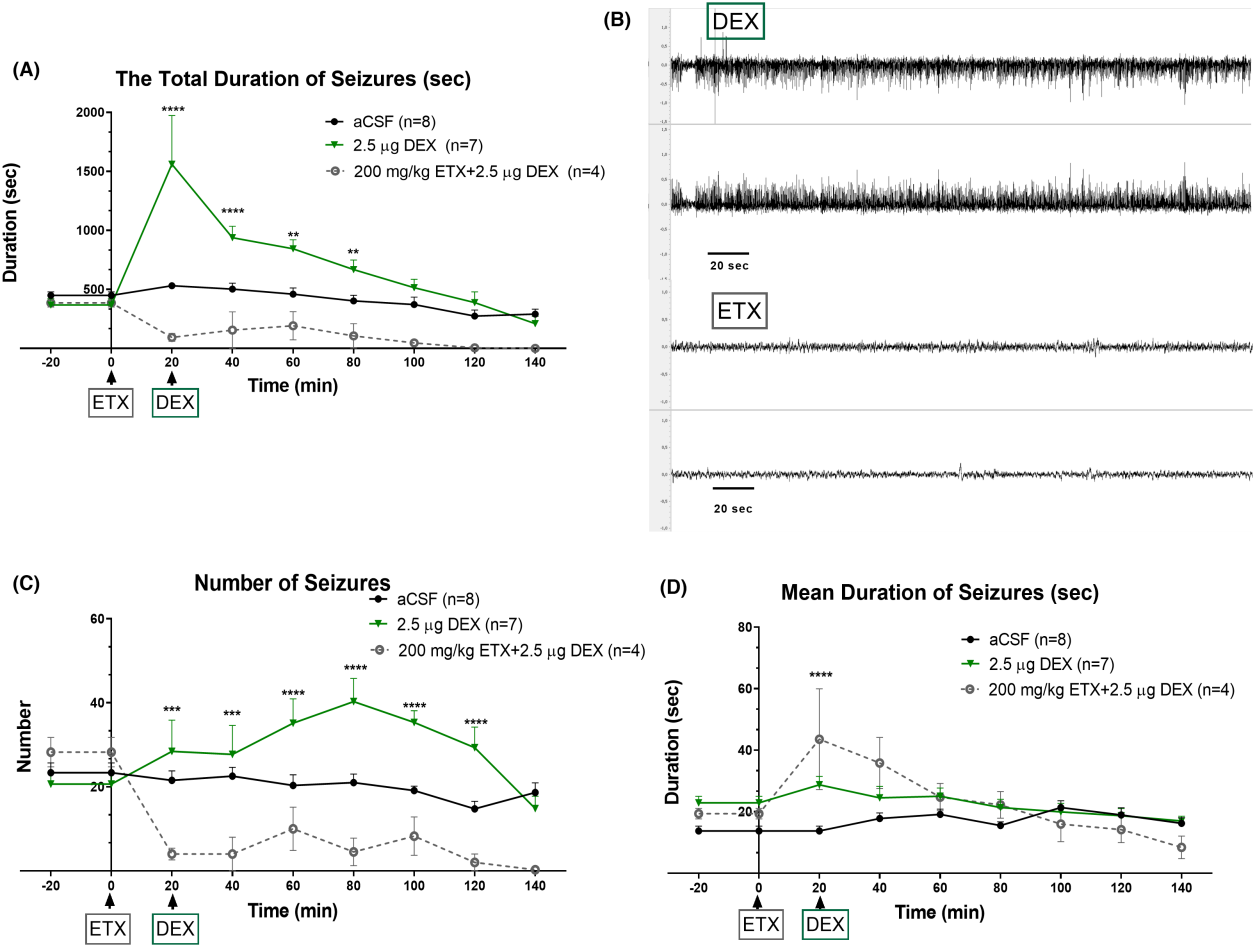
Pretreatment of ethosuximide suppressed the status events induced by 2.5 μg of DEX. DEX: Dexmedetomidine, ETX: Ethosuximide, aCSF: Artificial cerebrospinal fluid. (A) The mean total duration of SWDs in different doses of i.c.v. administered DEX. Data are given as means ± SEM (*p < 0.05; two‐way ANOVA followed by Tukey's post hoc test showed a significant increase with doses of 0.5 and 2.5 μg). Time 0 indicates the time of ethosuximide and 20 indicates the time of DEX injection. The number of rats is given in the legends. (B) ETX pre‐treatment suppressed DEX induced status events as seen in the EEG traces. (C) The number of SWDs in different doses of i.c.v. administered DEX. (D) The mean duration of one SWD complex, the ratio of total SWD duration to the number of SWDs (*p < 0.05; the significant increase by pretreatment of 200 mg/kg ethosuximide followed by 2.5 μg of DEX).

SWDs and absence status epilepticus is known to be responsive to ETX^34^ in humans. We investigated if pretreatment with 200 mg/kg, intraperitoneal ETX would prevent the absence status induced by DEX at the dose of 2.5 μg (i.c.v.). The ETX pre‐treatment completely prevented both the initial induction of absence status(es) as well as the second period of absence status(es) and decreased the overall SWD activity (Figure 4). ETX also reduced the total SWD duration and frequency in comparison to the aCSF applied group as expected (Total SWD Duration: “Time” effect: *F* (8, 150) = 5.660, *p* < 0.001; “Treatment” effect: *F* (2, 150) = 33.92, *p* < 0.001; Figure 3A) (“Time” effect: *F* (8, 150) = 3.639, *p* < 0.001; “Treatment” effect: *F* (2, 150) = 42.66, *p* < 0.001; Figure 5C). Still on the EEG of the rats occasional sleep spindles were seen as well as the rats were slightly sedated.

### 3.5 The effect of ETX, DIAZ, VPA, and ATI on the DEX‐induced events

**FIGURE 6:**
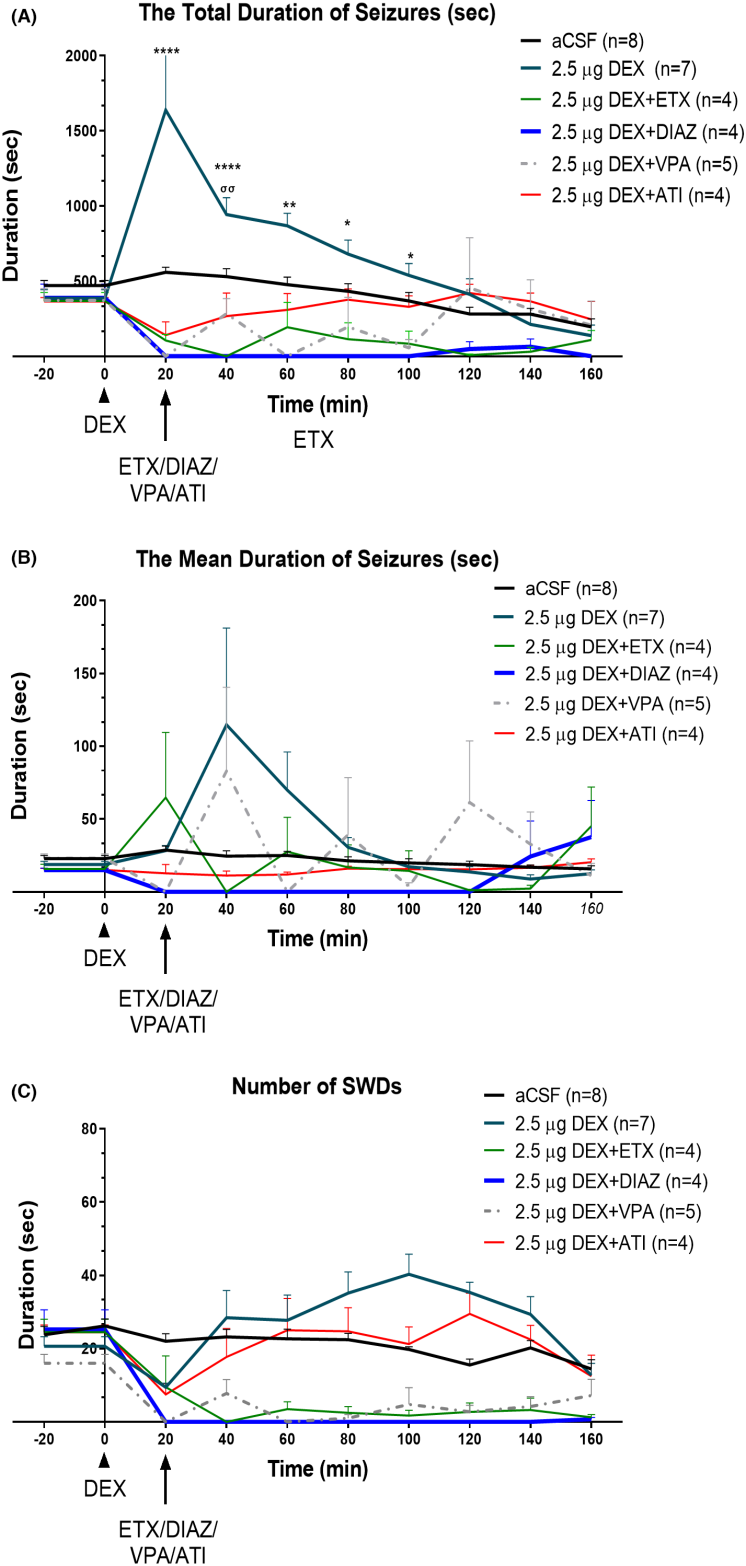
Post‐absence status treatment of ETX, DIAZ, VPA, and ATI on the DEX‐Induced Events. 2.5 μg DEX: Dexmedetomidine‐injected, 2.5 μg DEX + ETX: Post‐Ethosuximide injection after 2.5 μg DEX, during second set of absence statuses, 2.5 μg DEX + DIAZ: Post‐Diazepam injection after 2.5 μg DEX, during second set of absence statuses, 2.5 μg DEX + VPA: Post‐Valproic acid injection after 2.5 μg DEX, during second set of absence statuses, 2.5 μg DEX + ATI: Post‐Atipamezole injection after 2.5 μg DEX, during second set of absence statuses. (A) The mean total duration of SWDs. Data are given as means ± SEM (*p < 0.05; two‐way ANOVA followed by Tukey's post hoc test showed a significant increase with doses of 0.5 and 2.5 μg). The number of rats is given in the legends. (C) The number of SWDs (B) The mean duration of one SWD complex, the ratio of total SWD duration to the number of SWDs.

VPA and DIAZ are classical anti‐status medications.^35^ We investigated the intraperitoneal application of the ETX, DIAZ, VPA, and ATI upon the initiation of the second period of absence statuses. While ETX abolished the activities the most, upon reversal of sedation with VPA the mean duration of prolonged activities was back (40th and 120th min). Atipamezole on the other hand reversed the sedation while not completely abolishing SWDs, SWDs mean duration was expressed similar to the baseline (*p* > 0.05) (Figure 6).

All drugs suppressed the second period of absence status(es) and decreased the total duration and mean duration on the 20th, 40th, and 60th min. The total SWD duration and frequency in comparison with the aCSF applied group as expected (Total SWD Duration: “Time” effect: *F* (9, 262) = 3.71, *p* < 0.001; “Treatment” effect: *F* (5, 262) = 25.43, *p* < 0.001; Figure 6A) (“Time” effect: *F* (9, 278) = 10.22, *p* < 0.001; “Treatment” effect: *F* (5, 268) = 63.38, *p* < 0.001; Figure 6C). Still on the EEG of the rats occasional sleep spindles were seen as well as the rats were slightly sedated with ETX, DIAZ, and VPA, but not with ATI.

The mean duration of SWDs were decreased in all the groups but occasional prolonged activities were seen in ETX or VPA injected rats but not with DIAZ or ATI (Total SWD Duration: “Time” effect: *F* (9, 269) = 0.94, *p* < 0.001; “Treatment” effect: *F* (5, 269) = 2.40, *p* < 0.001; Figure 6A). Interaction of “time” and “treatment” was *F* (45, 269) = 2.19, *p* = 0.03.

### 3.6 Power spectrum and coherence analysis of pre‐ and post‐injection recordings

**FIGURE 7:**
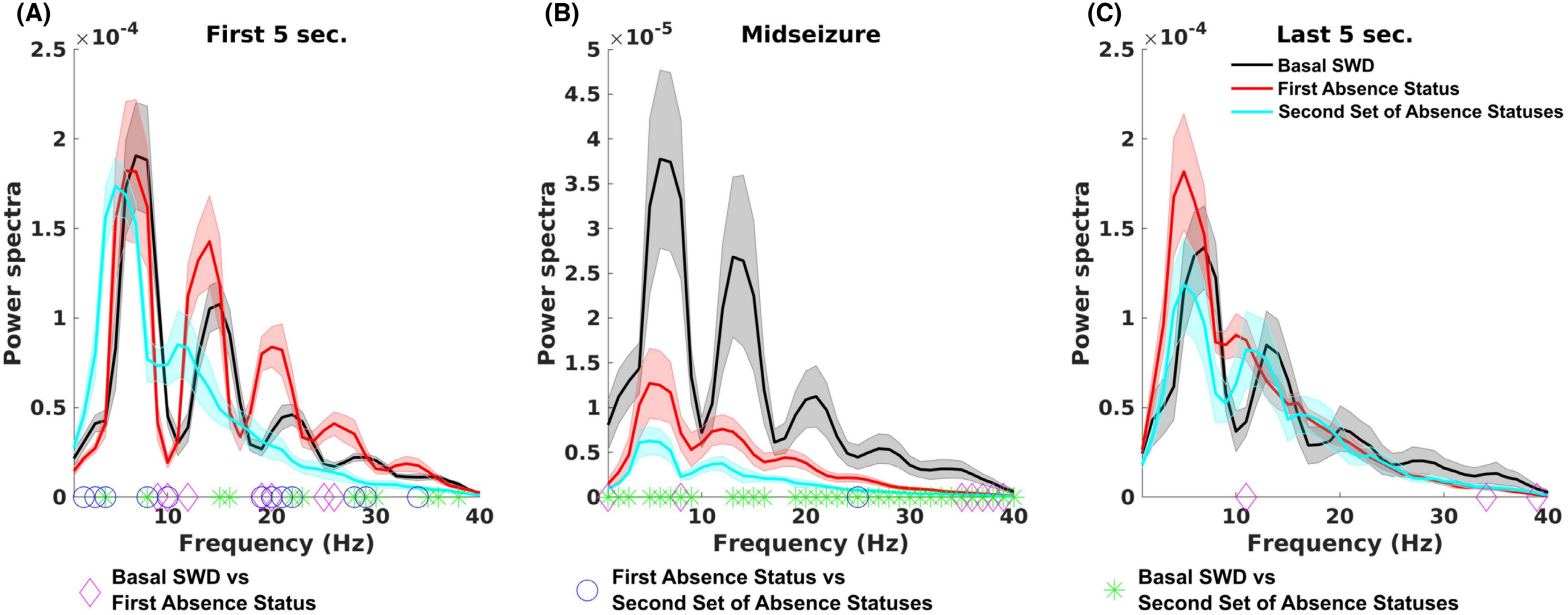
Power spectral density analysis. SWD: Spike‐and‐wave discharges. We compared the power amplitudes in seizures from each animal, *pre‐injection seizure* (basal SWD activities), *initial induction of absence status* (post‐DEX‐injection), and post‐sleep absence statuses (*the second period of absence‐status(es)*), which were analyzed in three periods, the first 5 s (A), the last 5 s (C), and the mid‐seizure phase (B). Signs show the frequencies where seizure types had statistical differences.

Power spectrum and coherence analysis were performed in the DEX‐2.5 μg group. Two rats were excluded from electrophysiological analyses due to electrical artifacts in the EEG recordings. PSD analyses of the mid‐seizure portion showed that the post‐sleep status event(s) the second period of absence status(es) and post‐injection status events (the initial induction of absence status) had lower power amplitude of 7 Hz and its harmonics (14 and 21 Hz) than the pre‐injection seizure (basal SWDs) (false discovery rate [FDR] corrected *p* < 0.05). PSD analyses also revealed that during the first 5 s of the second period of absence status(es) had higher power in delta frequency band (1–4 Hz) and attenuated power of 7 Hz harmonics (14 and 21 Hz) than the pre‐injection seizure (basal SWDs) and the initial induction of absence status. The first peak frequency in the second period of absence status(es) was slower than pre‐injection seizure and initial induction of absence status (5 Hz vs. 5.9 Hz vs. 6 Hz) during all periods of the events (Figure 7).

Coherence between the two hemispheres showed no difference between seizures (SWDs and status events) in the frequency range analyzed (FDR corrected *p* > 0.05).

Lastly, the variance of the peak voltages was different between seizures in five of six rats (*x* = 14.92–33.76, df = 2, FDR‐corrected *p* < 0.05). In four rats, the second period of absence status(es) had the highest variance of peak voltages.

## 4 DISCUSSION

This study established continuous SWD patterns with a specific α_2AR_ agonist DEX in GAERS. DEX‐induced experimental approach (DEX‐induced status in GAERS) may represent an acute absence status epilepticus model. Absence statuses were induced in 100% of rats with a specific dose of 2.5‐μg DEX, delivered i.c.v., in an already existing absence pathophysiology of the GAERS model, which is well described as one of the genetic absence epilepsy models in epilepsy research with the WAG/Rij.^36^, ^37^ In our previous study, we had administered the ATI to suppress the SWDs through the intracerebroventricular route and therefore here we used the same method of drug administration in this study as well. A previous study reported that continuous seizures were induced by mixed antagonists of D1/D2 receptors in GAERS or audiogenic model.^17^, ^38^, ^39^ The two phase of events that DEX induces which are an initial period of absence status‐like activity followed by sleep, then an immediate switch from sleep to the second period of absence status‐like events. The switch is immediate timewise. Therefore, further studies are planned to address to understand this time‐sensitive switch.

Conversely, noradrenaline is a known modulator of dopamine release, specifically by introducing specific *α*_2AR_ agonists in VTA, and *α*_2AR_ is shown to increase the phasic dopamine release in the ventral tegmental area.^40^ This study strengthens the hypothesis that α_2AR_ is one of the main mechanisms behind the absence seizures.^21^, ^26^ With our previous article and studies,^21^, ^41^ α_2AR_ is found to underline the pathophysiology of SWD activities. In the present study, the injection of a specific agonist DEX lengthened the mean duration of SWD events by converting the absence activity to absence status epilepticus. The increased mean duration of SWDs has been previously reported to be a result of stopping mechanism disturbances of SWDs and decreased GABAergic availability in the reticular thalamic nucleus.^42^, ^43^ However, the direct involvement of the dopaminergic system and how it is modulated by α_2AR_ requires further electrophysiological studies, possibly with knockout models.

DEX is a sedation agent that induces sleep.^44^ Absence epilepsy has especially been associated with non‐rapid eye movement (NREM) sleep due to shared physiological pathways.^45^ In our study, transitions between the status and sleep phases have brought the idea of transitional differences in thalamo‐cortical burst firings between NREM and absence seizures.^46^ NREM sleep allows the SWDs as compared to REM^47^; however, in our study, the sleep and status events were completely separate. Therefore, this model can also be a tool to investigate sleep and absence status transitions. The sedation sleep induced by DEX showed a character of deep sedation as explained previously.^24^ Interestingly, the post‐sleep seizure had higher power in a delta frequency band (1–4 Hz) and attenuated power in 7‐Hz harmonics (14 and 21 Hz) than pre‐ and post‐injection seizures. Moreover, the attenuated power in beta frequencies might also indirectly indicate the dopaminergic release as stated and discussed in previous studies.^48^

Haloperidol in this study shifted the beta frequency down in the prefrontal cortex of patients and monkeys with Parkinson's disease. In our study, it can signify the dopaminergic release in cortical areas due to α_2AR_ antagonism. To compare beta frequencies based on the harmonics during the first SWD frequency spectrum, data visualized that the beta power is also expected to be significant; however, this result cannot be interpreted.

We have demonstrated that the activity induced by the α_2AR_ DEX can be effectively blocked by ATI. Rather than simply suppressing the entire spike‐and‐wave discharges (SWDs), ATI instead reversed the activity, leading to the expression of SWDs in a normal manner (in terms of mean duration). VPA exhibited suppressive effects for up to 2 h, while diazepam induced sleep to eliminate the activity. Conversely, ETX still affected the rats' wakefulness. It appears that the α_2AR_ receptor may play a crucial role in the sustained periods of prolonged SWDs, and even VPA, known for its multi‐acting pharmacological properties, fails to fully suppress this phenomenon that would exclude other mechanisms of actions such as the blockage of voltage‐gated ion channels. With ETX, there was a variance between rats especially, with the mean duration. Besides there is no conventional use of ETX for the case of absence status epilepticus, one study reports an absence status incident when the child patient was on ETX, and a benzodiazepine agent was used to block the status.^49^ Also, there were occasional prolonged activities with VPA and ETX but not with DIAZ or ATI. ATI immediately woke rats, and on the EEG, there were no spindle activity were observed unlike with the other drugs. ATI caused a switch from the sleep and absence status to ordinary SWDs, unlike other drugs, without causing any sedation or lethargy.

Post‐injection absence statuses had lower frequencies (<2.5 Hz), which is slightly lower than the average SWD frequency compared to the absence status epilepticus.^4^, ^9^ Similarly, a higher variance complies with the absence status epilepticus definition in humans. Besides, the sensitivity of absence status epilepticus events toward ETX^34^ strengthens the representation of human absence status epilepticus. ETX is a classical anti‐absence drug, and its suppressive effects for SWDs have been shown in our studies.^50^, ^51^ Therefore, we did not want to use more than four rats in this study.

The most significant contribution of this study is that it proposes a research model of the pathophysiology of absence status epilepticus. Absence status epilepticus animal models are valuable since it is not easily defined or possibly overseen during the lifespan of patients. This model will enable future research to focus on the molecular level changes during the induction and continuation of this absence status epileptics and investigate possible micro‐damages during seizures. Furthermore, DEX should be cautiously used in inducing anesthesia in children with epilepsy.^52^

## 5 CONCLUSION

This study presents a model of acute absence status epilepticus induced by DEX in GAERS. A dose of >0.5 μg of DEX can induce absence statuses, and a 2.5 μg dose of DEX especially can induce strong absence statuses. This study strengthens both the role of α_2AR_ in the SWD generation and promotes the use of an acute model of absence epilepsy to preclinically evaluate drug therapies; investigate the possible pathophysiologies, damages, and comorbidities of the absence epilepsy; assess sleep and seizure transitions. Unveiling this switch mechanism with further studies would be very interesting in evaluating state transitions of sleep and SWDs. ATI seems to be a potential candidate for absence status epilepticus treatment.

## AUTHOR CONTRIBUTIONS

Melis Yavuz and Filiz Onat conceptualized and designed the study. Melis Yavuz organized the database, performed the statistical analysis, and wrote the first draft of the manuscript. Serdar Akkol performed the analysis and wrote the Results section of the PSD section of the manuscript. Melis Yavuz and Filiz Onat contributed to the manuscript's revision and read and approved the submitted version. All authors approved the final version of the manuscript.

## FUNDING INFORMATION

This work is **s**upported by ABAPKO [grant number 2022/02‐20] and Europe Commission [GEMSTONE Horizon Europe grant number 101078981]. Patent application number: 2021/014211 TPI.

## CONFLICT OF INTEREST STATEMENT

The authors declare that they have no conflicts of interest. We confirm that we have read the journal's position on issues involved in ethical publication and affirm that this report is consistent with those guidelines.

## Data Availability

The data are available upon request from the authors.
